# Activated Met Signalling in the Developing Mouse Heart Leads to Cardiac Disease

**DOI:** 10.1371/journal.pone.0014675

**Published:** 2011-02-09

**Authors:** Christian Leo, Valentina Sala, Mara Morello, Amedeo Chiribiri, Ilan Riess, Daniele Mancardi, Stefano Schiaffino, Carola Ponzetto, Tiziana Crepaldi

**Affiliations:** 1 Department of Anatomy, Pharmacology and Forensic Medicine, University of Turin, Turin, Italy; 2 Department of Internal Medicine, University of Turin, Turin, Italy; 3 Department of Clinical and Biological Sciences, University of Turin, Turin, Italy; 4 Venetian Institute of Molecular Medicine (VIMM), Padoa, Italy; Maastricht University, Netherlands

## Abstract

**Background:**

The Hepatocyte Growth Factor (HGF) is a pleiotropic cytokine involved in many physiological processes, including skeletal muscle, placenta and liver development. Little is known about its role and that of Met tyrosine kinase receptor in cardiac development.

**Methodology/Principal Findings:**

In this study, we generated two transgenic mice with cardiac-specific, tetracycline-suppressible expression of either Hepatocyte Growth Factor (HGF) or the constitutively activated Tpr-Met kinase to explore: i) the effect of stimulation of the endogenous Met receptor by autocrine production of HGF and ii) the consequence of sustained activation of Met signalling in the heart. We first showed that Met is present in the neonatal cardiomyocytes and is responsive to exogenous HGF. Exogenous HGF starting from prenatal stage enhanced cardiac proliferation and reduced sarcomeric proteins and Connexin43 (Cx43) in newborn mice. As adults, these transgenics developed systolic contractile dysfunction. Conversely, prenatal Tpr-Met expression was lethal after birth. Inducing Tpr-Met expression during postnatal life caused early-onset heart failure, characterized by decreased Cx43, upregulation of fetal genes and hypertrophy.

**Conclusions/Significance:**

Taken together, our data show that excessive activation of the HGF/Met system in development may result in cardiac damage and suggest that Met signalling may be implicated in the pathogenesis of cardiac disease.

## Introduction

The cellular events occurring during the early stages of life, including pre- and perinatal phases, may have strong impact on long-term health. Epidemiological and experimental evidences suggest that development of cardiovascular diseases in the adult is influenced by stressful events during late prenatal or early postnatal life [1]. A correlation between infant mortality and the incidence of cardiovascular disease was first reported in 1977 and lead to the Barker's hypothesis of the fetal origins of increased risk of cardiovascular disease [2]. The fetal origins hypothesis of Barker states that programming during fetal life occurs in response to an adverse environment and results in permanent adaptive responses that lead to structural and physiological alterations and the subsequent development of cardiovascular disease. Although this hypothesis was originally proposed in the context of intrauterine growth, it has been extended to the important environmental transition which occurs between plastic phase of development and mature post-plastic phase. In rodents, transition of cardiomyocytes from hyperplasia to hypertrophy growth occurs during the first week of postnatal period [3]. In parallel with this transition, murine cardiomyocytes accumulate contractile proteins and undergo changes in troponin I (TnI) and myosin heavy chain (MHC) isoform expression. The cardiac TnI (cTnI) and the slow skeletal TnI (ssTnI) transcripts coexist in the developing heart throughout fetal and perinatal stages and then cTnI completely replaces ssTnI in the adolescent mouse [4], [5]. Concurrently, α-MHC completely replaces β-MHC in the ventricles, becoming the dominant isoform (>90%) in the adolescent mouse [6]. Besides myofibrillar content, important cell-shape changes occur in cardiomyocytes during early postnatal development, with progressive polarization of the cardiomyocyte and restriction of the intercalated disc-associated proteins to the bipolar ends of cardiomyocytes [7].

The Hepatocyte Growth Factor (HGF) is a mesenchyme-derived multifunctional molecule that elicits mitogenic and morphogenic activities in development, as well as in many patho-physiological processes [8]. The HGF receptor has been identified as the Met tyrosine kinase, the product of the *met* proto-oncogene, which is expressed in a variety of cell types, such as epithelial, endothelial and mesenchymal cells. Upon HGF binding, Met undergoes autophosphorylation on several tyrosine residues and constitutes a multifunctional-docking site for adaptor proteins containing the SH2 motif. Recruitment of these molecules results in the activation of several downstream signalling cascades, such as Ras-Raf-MEK-ERK and PI3K pathways, which are essential for HGF-induced cellular changes, that collectively give rise to a complex morphogenetic program known as “*invasive growth*”. This program involves cell spreading, cell-cell dissociation, migration, invasion, proliferation and differentiation. Notably, during the various phases of this process, cells result protected from apoptosis [9].

In the heart, HGF has been shown to exert anti-apoptotic/cardioprotective effects in rats subjected to myocardial infarction [10], [11]. It has also been suggested to have a role in cardiac regeneration after myocardial infarction [12]. Met is only weakly expressed in adult cardiomyocytes, but both HGF and Met mRNA are induced following heart injury [10], [11]. The beneficial effect of HGF in the damaged heart has been documented in experimental models. Despite the clear indications for HGF to effectively treat post-ischemic heart failure, the knowledge on the role of the HGF/Met system in normal cardiac development is still limited. A few reports have shown expression of HGF/Met in the heart during very early embryonic development [13], [14], whilst studies investigating their expression and function in the heart during late prenatal or early postnatal life are still missing. In particular, nothing is known about the role of the HGF/Met system in the establishment and maintenance of the balance between proliferative and differentiating events during postnatal heart development, which may lead to hyperplastic and hypertrophic growth, respectively.

In this study, we aimed to investigate this issue by activating the HGF/Met system specifically in the heart. To this purpose we generated two novel gain-of-function transgenic models with tetracycline-suppressible expression of either HGF or activated Met under control of the α-MHC promoter. In the mouse embryo, the α-MHC promoter is expressed throughout the myocardium starting from E8 [6]. By E12.5, α-MHC is robustly expressed in both left and right atria. However, transcripts are more abundant in the right than the left ventricle. Expression in the right ventricle is also downregulated with respect to that in the atria. At E14.5 and E16.5, α-MHC gene is strongly expressed in both atria, while in the ventricles it retains a right dominant profile. At birth, α-MHC transcript levels start increasing and, by postnatal day 3, α-MHC is upregulated in both the right and the left ventricle [15]. Thus, the α-MHC promoter is especially useful for analyzing the effect of transgenic protein during the prenatal and postnatal period of rapid heart growth. In the HGF model, autocrine production of HGF stimulated the activation and physiological downregulation of endogenous Met receptor. In the model of Met activation, the intracellular signal stemmed from ligand-independent and constitutive stimulation of Met kinase in cardiomyocytes. We show that even transient increases in HGF/Met signalling during development can lead to cardiac pathology, due in part to sustained downregulation of Cx43.

## Materials and Methods

### Ethics Statement

All animal procedures were approved by the Ethical Commission of the University of Torino, Italy, and by the Italian Ministry of Health, both of which accepted the use of mice for this study (A/R 0045 and A/R 0041).

### Conditional cardiac HGF tg mice

The mouse HGF cDNA was cloned into the pBI-EGFP plasmid which is responsive to tTA transactivator [16]. The construct was linearized with AseI and a 6.1-kb gel-purified fragment was microinjected into the fertilized eggs of FVB mice in the San Raffaele-Telethon Core Facility for Conditional Mutagenesis (Milan, Italy). Founder mice were identified as described [17]. A transgenic line (HGF-TRE-GFP responder) was bred with the α-MHC-tTA mouse (kindly donated by Dr. G. Fishmann [18]) and double heterozygotes were studied under one of three conditions: (1) Mice were never administered Doxycycline (DOX, Sigma), so that HGF was expressed in the prenatal and postnatal period (HGF tg mice). (2) Pregnant mothers and suckling progeny received DOX (200 µg/ml in drinking water with 3% sucrose) to continuously repress HGF (HGF + DOX tg mice). (3) Pregnant mothers were not administered DOX to induce HGF *in utero* and suckling progeny and weaned pups received DOX to repress exogenous HGF after birth (prenatal HGF tg mice).

### Conditional cardiac Tpr-Met mice

The Tpr-Met-TRE-GFP responder mouse [17] was bred with α-MHC-tTA mouse and double heterozygotes were studied under one of two conditions: (1) Mice were never administered DOX (Tpr-Met mice). (2) Pregnant mothers received DOX throughout gestation and DOX was removed at P1 to induce Tpr-Met after birth (postnatal Tpr-Met mice). Control mice consisted of identically treated littermate wild-type mice for both transgenic models.

### Real-time PCR and Semi-quantitative RT-PCR

Hearts were excised, rinsed in ice-cold Tyrode solution and prepared in RNA later (Ambion). Total RNA was extracted with TRIzol (Sigma). Qiagen RNAeasy kit (Qiagen GmbH, Hilden, Germany) was used to enhance purification. After quantification (NanoDrop® ND-1000, NanoDrop Technologies), reverse transcription was performed using DNA Polymerase/Superscript III Reverse Transcriptase (Invitrogen). For Real-time PCR, primers and Taqman probe specific for the transgene were designed using the File builder 3.1 program (Applied Biosystems, Foster city, CA, USA). Real-time PCR was performed on a 7300 Real-time PCR instrument (Applied Biosystem). Sample reactions were performed in triplicate and normalized to 18S mRNA expression. For semi-quantitative RT-PCR, control samples were prepared without adding the RT enzyme to the reaction. Tubulin was used as control. See Table S1 for primers used.

*Western blot* - Protein extracts from heart ventricles were prepared using RIPA buffer added with Protease Inhibitor Cocktail (Sigma). Heart lysates were subsequently sonicated and centrifuged at 14000rpm (25′ at 4°). Protein concentration was determined by Bio-Rad protein assay. Protein lysates (5 µg or 100 µg for Met protein) were separated by SDS-PAGE, transferred to nitrocellulose membrane Hybond-C-extra (Amersham) and blotted with primary antibodies and then with horseradish peroxidase-conjugated secondary antibodies (Amersham). Proteins were revealed by enhanced chemiluminescence SuperSignal detection reagents (Pierce) and quantified with GS800 model Bio-Rad (Figures 3,4) and ImageJ (rsb.info.nih.gov/ij) (Figures 2,6).

### Stereomicroscopy, Immunofluorescence (IF) and Confocal Analysis

Hearts were removed, rinsed in ice-cold Tyrode solution and fixed in 4% paraformaldehyde (PAF) in phosphate-buffered saline (PBS) for 4 hours at 4°C. After PBS washings, hearts were incubated in 30% sucrose in PBS overnight at 4°C to preserve GFP fluorescence. Stereomicroscopy was viewed by Leica MZ12 and imaged by Evolution VF colour cool camera and Image-Pro Plus software. Tissues for indirect immunofluorescence were embedded in OCT (Biooptica), quickly frozen in isopentane and stored at −20°C. Sections were 20 µm cut, post-fixed 5′ in ice-cold 4% PAF and washed with PBS. For Met staining, sections were incubated with SP260 primary antibody (Santa Cruz) overnight at 4° and subsequently with Alexa Fluor 546-conjugated goat anti-rabbit antibody (Molecular Probes) for 1h at room temperature. The double overlay pictures (Met/GFP) were viewed with a Leica DM6000 CS confocal microscope. Optical slices (1024 by 1024 pixels, frame resolution) were acquired at 10Hz and processed with LAS AF software (Leica Microsystems CMS GmbH). Quadruple overlay pictures (Laminin/Griffonia/DAPI/GFP) were obtained by staining with rabbit polyclonal antibody laminin (Sigma) followed by Alexa Fluor 647-conjugated goat anti-rabbit antibody (Molecular Probes), two hours incubation with rhodamine Griffonia Simplicifolia (Vector Laboratories) and 5 min with DAPI. Quadruple overlay pictures (Cx43/α-actinin/DAPI/GFP) were obtained by staining with rabbit polyclonal antibody Cx43 (Sigma) and mouse monoclonal antibody α-actinin (Sigma) and subsequently with Alexa Fluor 647-conjugated goat anti-rabbit antibody and Alexa Fluor 546-conjugated goat anti-mouse antibody (Molecular Probes). Confocal microscope imaging was performed with Leica TCS SP2 AOBS upright microscope. Optical slices (1024 by 1024 pixels, frame resolution) were acquired at 200 Hz, with a line average of 8, and processed with LAS AF software (Leica Microsystems CMS GmbH).

### Proliferation evaluation

Ki67 positive nuclei were immunostained in 20 µm thick heart sections. Primary rabbit polyclonal Ki67 antibody (Novocastra) and secondary Alexa Fluor 546-conjugated goat anti-rabbit antibody (Molecular Probes) were used. Fluorescence imaging and processing were performed with Leica DM6000 CS confocal microscope and LAS AF software, respectively. 5 fields per area (Right Ventricle, Left Ventricle and Interventricular Septum) per mouse were analyzed. 3 mice per group were considered.

### H9c2 cell proliferation assay

H9c2 cell line purchased from the American Type Culture Collection was grown as described [19]. 4000 cells/cm^2^ were seeded. Adhesion medium was replaced with fresh medium containing 10 U/ml of HGF for 24h and 48h. AlamarBlue™ (Invitrogen) assay was performed according to manufacturer's instructions. For BrdU assay, cells were seeded on coverslips and incubated with 10 µm BrdU (Sigma) for 24h, together with treatment. Cells were fixed with 4% PAF, permeabilized with 0.1% tween, treated with 2 M HCl for 1h and stained with BrdU-specific antibody (Sigma) and Alexa Fluor 488-conjugated goat anti-mouse antibody (Molecular Probes). Propidium iodide was used for nuclear staining. Fluorescence imaging and processing were performed with Leica DM6000 CS confocal microscope and LAS AF software, respectively. All images were taken with the same parameters of exposition and processed after conversion in 8bit grayscale. Stained nuclei were counted using ImageJ. A minimum cut-off for intensity and particle size was established. Nuclei on border edges were excluded. 7 fields per sample and 2 replicates were considered.

### Antibodies

See Supplemental Table S2 for a list of antibodies used.

### Lucifer yellow Assay

Gap junction permeability assay was performed as described [20] with minor modifications. The ventricle was incubated in buffer containing Lucifer yellow (2.5 mg/ml) and rhodamine-conjugated dextran (2.5 mg/ml), which was continuously bubbled with O_2_, for 20′ at 37°C. Then, the ventricle was fixed with 4% PAF. The area stained with Lucifer yellow but not with Rhodamine red was used as an index of gap junction communication. Short incubation and bubbling with oxygen were performed to prevent anoxic effects on cellular permeability. Images were obtained by classical microscopy analysis (Leica DMRE microscope) of both ventricles. Data from 15 data sampling were averaged for each of 4 fields per mouse. 3 mice per group were analyzed.

### Histological Analysis and Fibrosis Evaluation

Hearts were rinsed in PBS, dehydrated and embedded in paraffin. Sections (6–8 µm thick) were rehydrated, stained with hematoxylin-eosin or Masson's trichrome and analyzed with Leica DMRE microscope.

### CSA

3 mice per group were analyzed. Transversal 20 µm thick cryo-sections of the middle region of the hearts were stained with rhodamine Griffonia Simplicifolia and rabbit polyclonal antibody laminin and subsequently with Alexa Fluor 488-conjugated goat anti-rabbit antibody (Molecular Probes) and DAPI. Fluorescence images were taken at 40× magnification with Leica DM6000 CS confocal microscope and LAS AF software was used for processing. 15 cross sectional areas of 6 fields per heart were measured. Small, medium and large-sized fibers were equally considered. Fiber CSA delimited by laminin staining was measured using ImageJ software. Density probability distribution curves were generated.

### Cx43 quantification

Images were obtained by classical microscopy analysis (Leica DMRE microscope) of ventricles and interventricular septum at 20×. Data from 10 samplings were averaged for each heart. 6 controls and 3 transgenics were analyzed. Signal intensity of staining was calculated as percentage of total tissue area using ImageJ.

### Echocardiography

Size and function of the left ventricle of the mice were evaluated by high-resolution echocardiography. M-mode examinations were performed using a dedicated small-animal high-resolution imaging unit (Vevo 770; VisualSonics, Toronto, Canada) and a 40-MHz high-frequency linear transducer (RMV 707B; VisualSonics, Toronto, Canada). Mice were kept anesthetized with tribromoethanol (Avertin, 350 mg/kg). Real-time imaging was performed with a frame rate of 100 Hz (temporal resolution of 10 msec). The following parameters were measured: systolic and diastolic thickness of the interventricular septum, end-systolic (LVESD) and end-diastolic diameter (LVEDD) of the left ventricle, systolic and diastolic thickness of the posterior wall of the left ventricle. Fractional shortening (FS) was then calculated [21]. The hypertrophy index h/r ratio was calculated according to the formula:
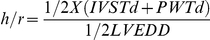
All measurements were done on 3 consecutive beats with a stable heart rate >400 bpm.

### Statistics

Data are expressed as the mean ± SD. Differences between groups were determined by independent T-tests (one or two-tailed T-tests have been used; details in each Figure Legend).

## Results

### Generation and Characterization of HGF tg Mice

**Figure 1:**
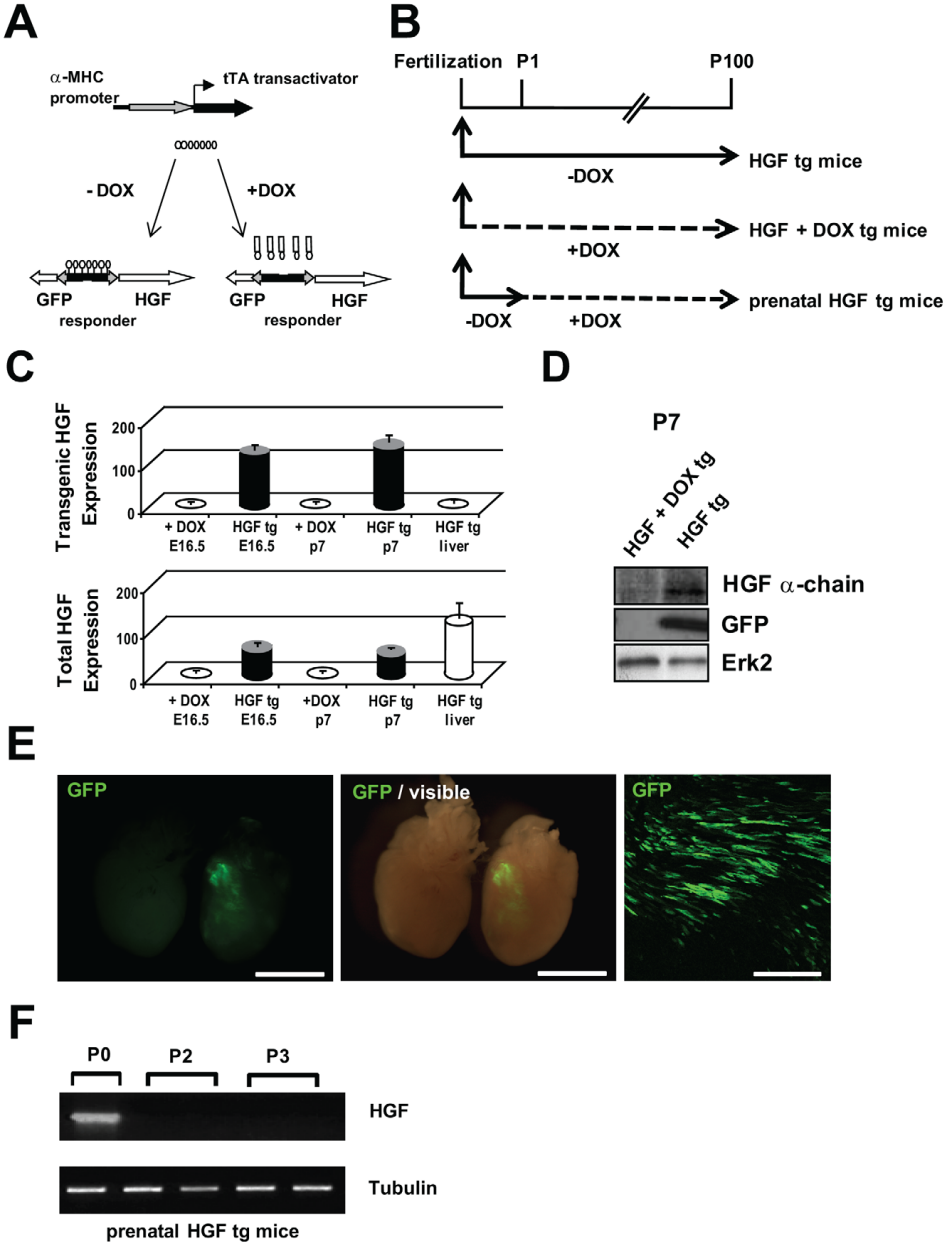
Doxycycline (DOX)-suppressible expression of HGF in the heart. (A) Schematic representation of the two components (α-MHC-tTA and HGF-TRE-GFP transgenes) for bitransgenic conditional HGF expression. (B) Top: Experimental design: mice were never administered DOX to express HGF in the prenatal and postnatal period (HGF tg mice); pregnant mothers and suckling progeny received DOX to continuously repress HGF (HGF + DOX tg mice); pregnant mothers were not administered DOX to induce HGF *in utero* and suckling progeny and weaned pups received DOX one day after birth to repress HGF in the postnatal period (prenatal HGF tg mice). (C) Quantitative Real-time PCR of exogenous (upper graph) and total (endogenous + exogenous) (lower graph) HGF mRNA in fetal (E16.5) and neonatal (P7) heart samples of bitransgenic mice, conceived in the presence or in the absence of DOX (n = 3 biological replicates). Liver tissue from HGF tg P7 mice was used as a control for the specificity of primers. (D) Representative Western blot of heart lysates of HGF tg mice with anti-HGF and anti-GFP antibodies. HGF protein migrates as the characteristic mature α chain of 70 kDa. Erk2 is the loading control. (E) Stereomicroscope images of GFP green fluorescence in neonatal hearts (P7) isolated from HGF + DOX (left) and HGF tg (right) mice: fluorescence (left panel) and visible plus fluorescence (middle panel); longitudinal tissue section showing fluorescent GFP+ cardiomyocytes in P7 HGF tg mice (right panel). Bars: 2.5mm (left panel); 250 µm (right panel). (F) mRNA expression of exogenous HGF in prenatal HGF tg hearts at different post-birth ages.

To examine the influence of HGF in normal prenatal and postnatal cardiac growth, we generated a bitransgenic α-MHC-driven tetracycline-suppressible system (Figure 1A). The GFP reporter was chosen as being a convenient tracer of transgene expression. Bitransgenic mice were conceived and maintained in the absence of Doxycycline (-DOX) to induce exogenous HGF expression (HGF tg) during prenatal and postnatal heart growth (Figure 1B). In parallel matings, pregnant mothers were given DOX starting from conception, throughout pregnancy and breastfeeding period (HGF + DOX tg). qRT-PCR was performed on exogenous and total (endogenous + exogenous) HGF mRNA in prenatal (E16.5) and neonatal (P7) hearts from uninduced (HGF + DOX tg) and induced (HGF tg) mice. Exogenous HGF mRNA could be detected only in HGF tg mice kept without DOX, but not in DOX-treated animals, both at E16.5 and at P7. Liver tissue was used to confirm specificity of primers for HGF transgenic form (Figure 1C, upper graph). Since primers for total HGF recognize both the wild-type and the transgenic forms, the quantification for uninduced mice refers specifically to endogenous HGF, while data from induced mice must be observed also considering transgenic HGF expression. Figure 1C shows that endogenous HGF was undetectable at embryonic day 16.5 and was still absent at postnatal day 7. Liver from neonatal HGF tg mice provided the positive control (Figure 1C, lower graph). Measurement of protein levels of HGF and GFP confirmed that the transgenes were not expressed in HGF + DOX tg animals, indicating a tight control of Tet-Off system of expression (Figure 1D).

Neonatal heart of HGF tg showed specific GFP expression (Figure 1E, middle and left panels; control at left and HGF tg at right). Expression of the transgene was heterogeneous in cardiomyocytes and variable between sibling mice, ranging from 20 to 50% of the cardiomyocytes in the left ventricle (Figure 1E, right panel). Bitransgenic HGF tg mice were born with the expected mendelian ratio and showed phenotypically normal hearts (Figure 1E and Figure S1).

In a cohort of animals (prenatal HGF tg mice), DOX was administered to suckling progeny 1 day after birth (Figure 1B). The HGF mRNA was repressed already after 1 day of DOX treatment (Figure 1F), indicating effective reversibility of the Tet-Off system.

### Met Is Present in Neonatal Cardiomyocytes and Exogenous HGF Activates its Downstream Effectors

**Figure 2:**
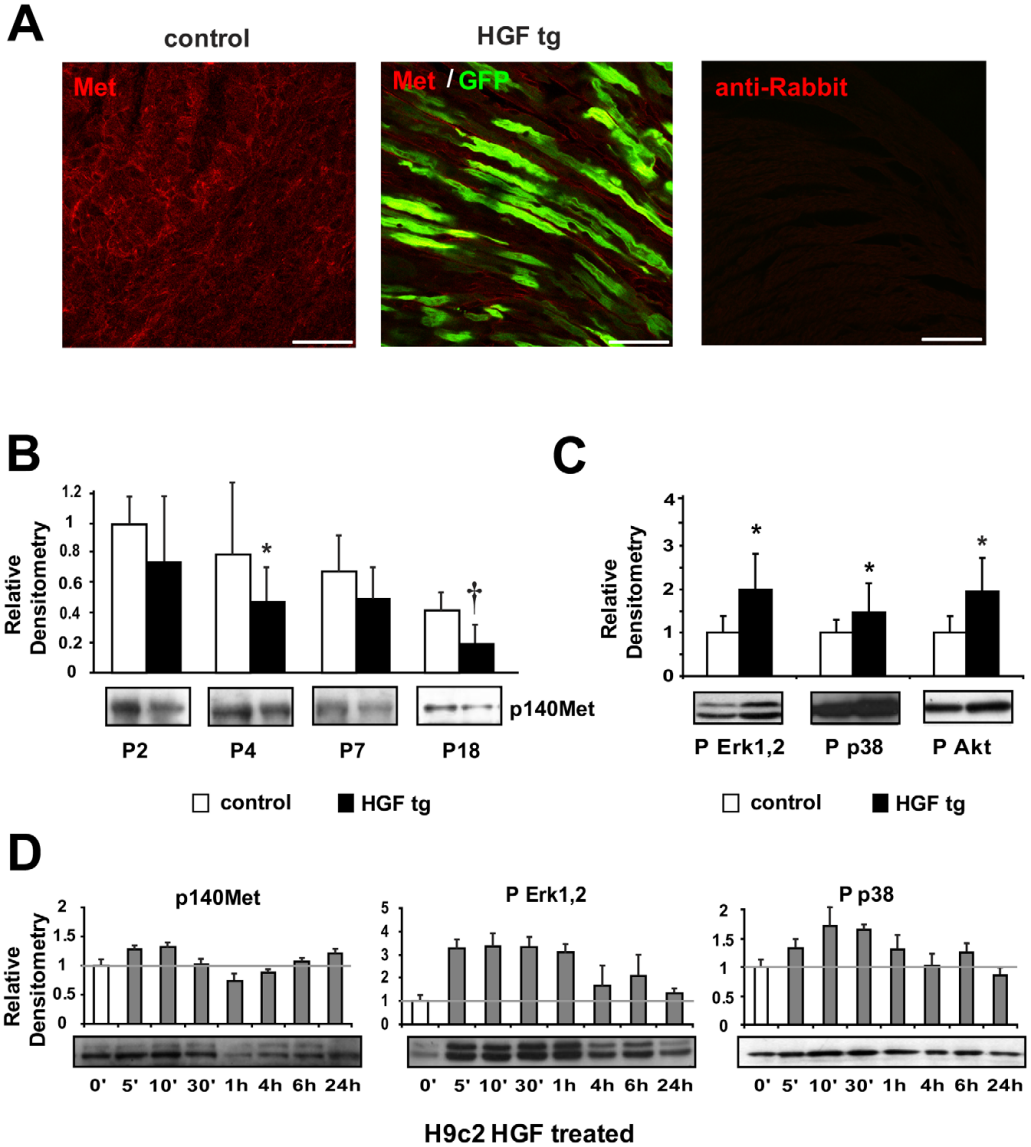
Analysis of Met expression and downstream signalling activation in neonatal cardiomyocytes. (A) Immunofluorescence of Met receptor (red) and GFP (green) in neonatal (P7) heart samples of control (left panel) and HGF tg mice (middle panel). A negative control of secondary antibody was included (right panel). Bars: 50µm. (B) Western blot of Met (p140Met) protein in control and HGF tg mice at different ages post-birth (P2 n = 6 n = 7, P4 n = 8 n = 6, P7 n = 10 n = 11, P18 n = 9 n = 14). Representative blots are shown below densitometric quantification (normalized on GAPDH loading control, relative to P2 control). Controls vs HGF tg mice: *p<0.05 and †p<0.005 (two-tailed T-test). (C) Densitometric quantification (normalized on tubulin loading control) and representative Western blot of phospho-Erk1,2 (P Erk1,2), phospho-p38 MAPK (P p38) and phospho-Akt (P Akt) in HGF tg (n = 7) relative to control mice (n = 6) at two days post birth (P2). *p<0.05 (two-tailed T-test). (D) Western blot analysis of Met receptor and downstream signalling after treatment of H9c2 cardiomyoblast cell line with 10U/ml of HGF for different lengths of time. Densitometric quantification was normalized against tubulin and plotted as relative to time 0′ of treatment. Each condition was tested 3 times.

Endogenous Met was localized all around the plasma membrane of cardiomyocytes in heart tissue isolated from littermate wild-type animals (control) and, at lower levels of expression, in HGF tg neonates (Figure 2A). The Met 140-kDa product (p140Met), clearly detectable in control neonates at P2 and P4, was downregulated at P7 and P18 (Figure 2B). In HGF tg mice, the level of Met was lower with respect to controls since P2 and it was further downregulated in adolescent mice at P18. Next, we evaluated whether exogenous HGF activated Met signalling in neonatal cardiomyocytes (Figure 2C). We found that in HGF tg mice there was a marked increase in Erk1,2, p38 MAPKs and Akt phosphorylation with respect to controls (p<0.05). We also tested the levels of expression and activation of Met signalling in the H9c2 cardiomyoblast cell line upon addition of recombinant HGF for various lengths of time (Figure 2D). After 1h of HGF stimulation, the Met receptor was downregulated. Its level of expression was recovered after 4h. Erk1,2 and, at lesser extent, p38 phosphorylation was stimulated 5′ after the addition of HGF and remained activated until 1h.

### Exogenous HGF Modulates Proliferation and Expression of Sarcomeric Proteins and Connexin43 in Neonatal Heart

**Figure 3:**
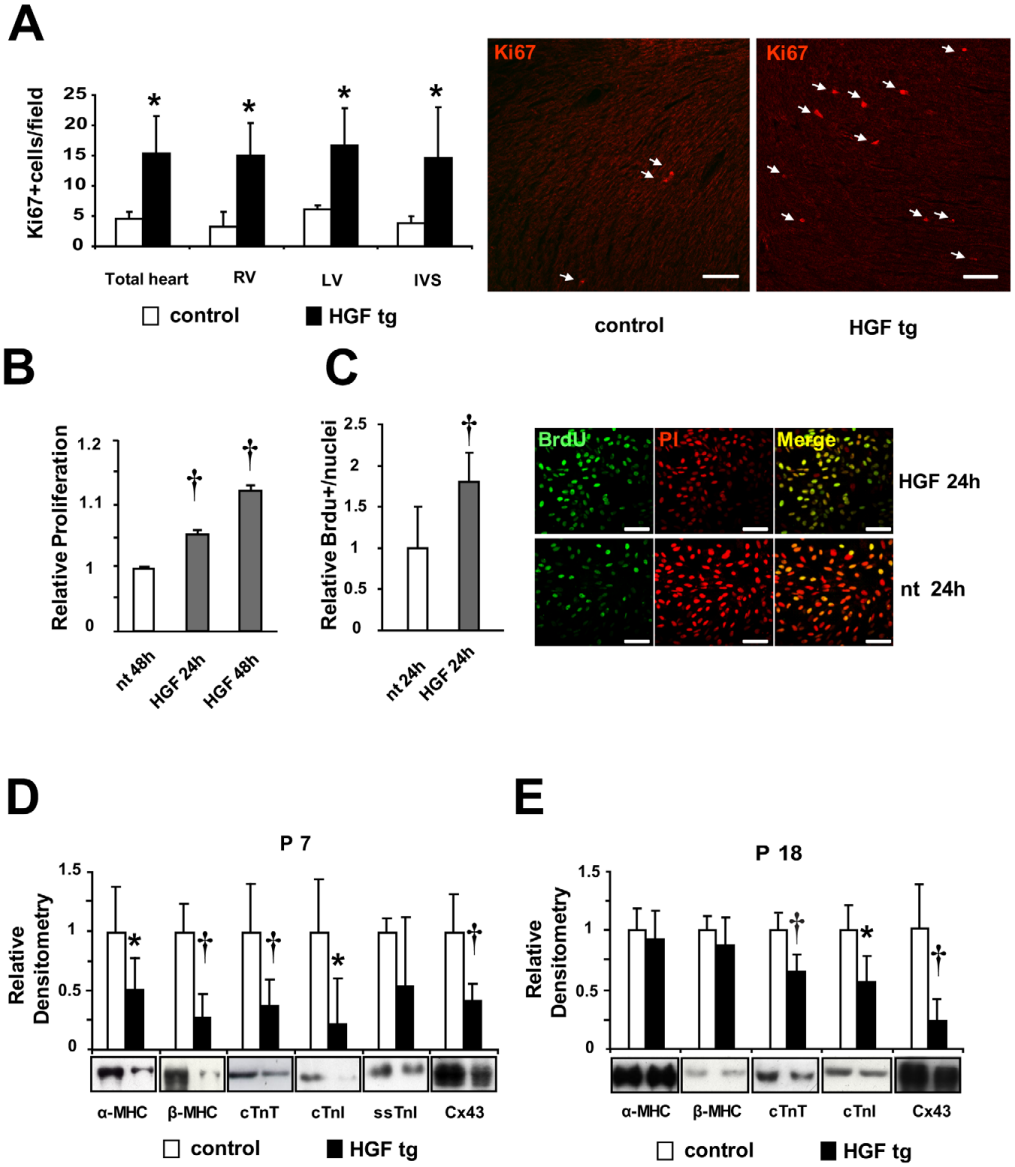
Increased proliferation and reduced expression of sarcomeric proteins and Connexin43 (Cx43) upon HGF induction in prenatal hearts. (A) Left panel: Quantification of Ki67 positive nuclei in tissue sections of 7 days-old neonatal hearts. Right Ventricle (RV), Left Ventricle (LV) and Interventricular Septum (IVS) were separately or totally analyzed and compared in controls vs HGF tg neonates (n = 3 animals per group). At least 5 fields per zone per sample were counted. *p<0.05 (one-tailed T-test). Right panel: Representative Ki67 staining in tissue sections of 7 days-old neonatal hearts of control (left) and HGF tg (right). Ki67: red-nuclear (white arrows). Bar: 100 µm. (B) AlamarBlue assay and (C) BrdU incorporation of H9c2 cell line not treated (nt) and treated with 10U/ml HGF for the indicated times. Experiments were done in 8 (B) and 2 (C) biological replicates for each sample group. †p<0.005 versus nt (two-tailed T-test). Right panels: representative IF. BrdU: green-nuclear; propidium iodide (PI): red-nuclear. Bar: 75 µm. (D,E) Densitometric quantification normalized to Erk2 loading control and representative Western blots of the indicated proteins. Results represent averaged values for immunoblot analyses performed on heart lysates in (D) P7 neonatal controls (n = 10) vs HGF tg (n = 11) and (E) P18 young adult controls (n = 9) vs HGF tg (n = 14). Myosin heavy chains (α and β-MHC), troponins (cTnT, cTnI and ssTnI) and Cx43 have been quantified. *p<0.05, †p<0.005 (two-tailed T-test).

To examine whether the extra-dose of HGF was able to increase proliferation of cardiac cells, we analyzed Ki67 positive cells in tissue sections of 7 days-old neonatal hearts. We found a 3-fold increase (p<0.05) in HGF tg mice, compared to controls (Figure 3A). To confirm that Met receptor stimulation promotes cardiomyocyte proliferation, we treated the cardiomyoblast cell line, H9c2, with 10U/ml recombinant HGF in vitro and analyzed cellular proliferation by means of AlamarBlue assay (Figure 3B) and BrdU incorporation (Figure 3C). H9c2 cell proliferation significantly increased after HGF treatment (p<0.005).

In parallel, we analyzed the levels of sarcomeric proteins in newborn mice. We observed that the levels of cTnT (p<0.005) and cTnI (p<0.05) were significantly lower in hearts of HGF tg neonates, as compared to controls (Figure 3D). ssTnI was also reduced, but the difference between HGF tg and controls was not significant (Figure 3D). In the adolescent HGF tg mouse, both cTnT and cTnI proteins were still downregulated (Figure 3E, p<0.005 and p<0.05, respectively), but reached normal levels in the adulthood (data not shown). At P18 ssTnI could not be detected in controls nor in HGF tg mice (not shown). The levels of both α- and β-MHC were decreased in HGF tg neonates, as compared to controls (Figure 3D, p<0.05 for α-MHC; p<0.005 for β-MHC). In the adolescent mouse, α-MHC completely replaced β-MHC, becoming the predominant isoform both in control and in HGF tg mice (Figure 3E), as described in literature [6]. We also evaluated the expression levels of the Cx43 protein, a marker of the working myocardium. In the neonate as well as in the adolescent, Cx43 protein levels were lower in HGF tg mice, compared to controls (Figure 3D,E, p<0.005).

### HGF Induction in Prenatal Heart Causes Reduced Cardiac Contractility in the Adult

**Figure 4:**
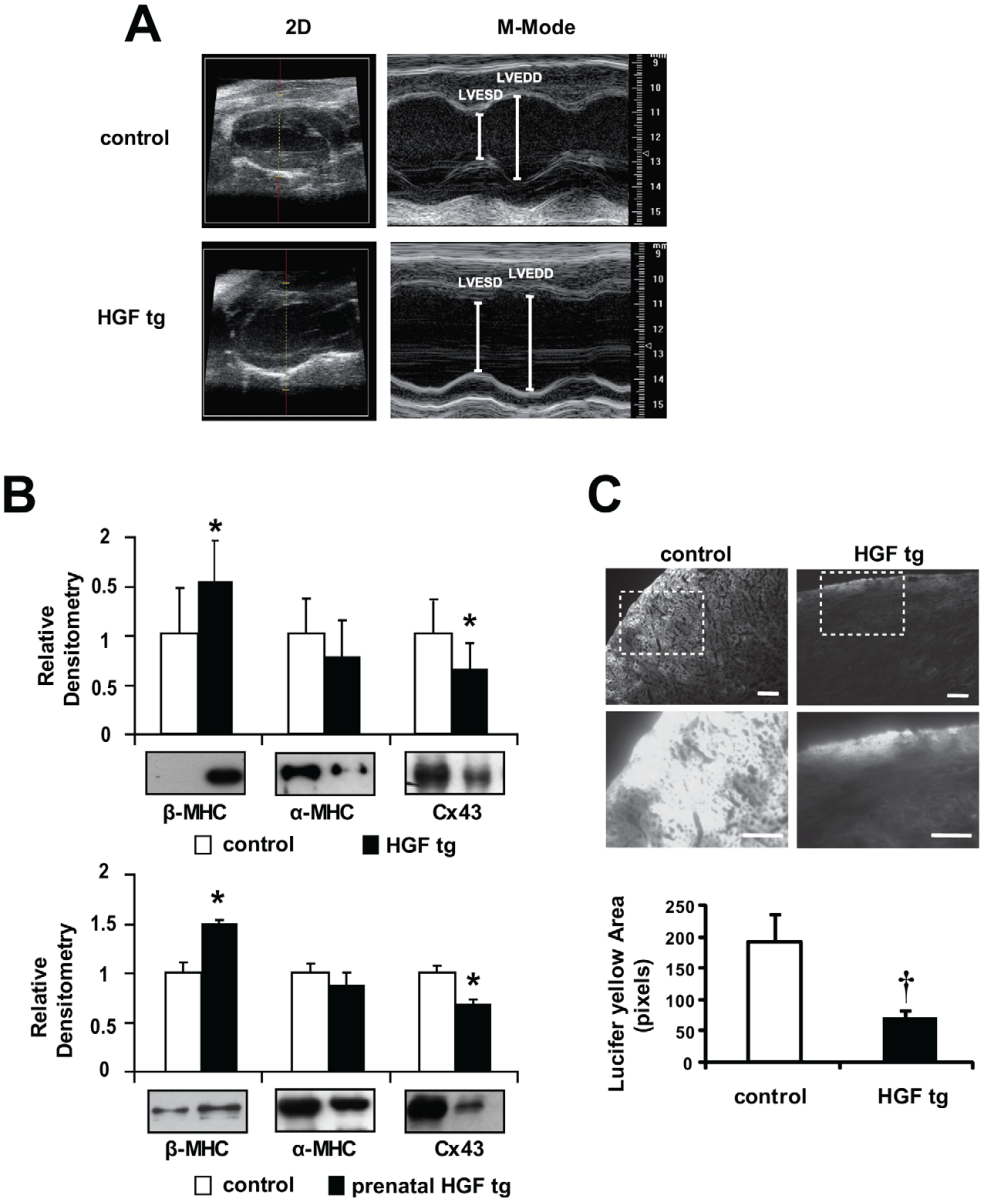
Contractile dysfunction, β-MHC re-expression and decreased Cx43 and cell-cell communication in adult HGF tg and prenatal HGF tg mice. (A) Representative images of Left Ventricle long-axis echocardiogram (2D and M-Mode) of control (upper panels) and HGF tg mice (lower panels). (B) Densitometric quantification normalized to Erk2 loading control and representative Western blots of heart ventricles from control vs HGF tg (upper graph) n = 9 mice per group and prenatal HGF tg (lower graph) n = 3 mice per group. In the latter, HGF expression was suppressed after birth. Re-expression of β-MHC and decreased Cx43 are evident in both bitransgenic mice compared to controls. *p<0.05 (two-tailed T-test). (C) Representative images of Lucifer yellow dye diffusion in HGF tg and control edge-cut hearts (upper panels) and zoom-in of the areas included in dashed boxes (lower panels). Bottom graph: quantification of pixel area showed that cell-to-cell spread of Lucifer yellow was significantly decreased in HGF tg mice vs controls (n = 3 mice per group). †p<0.005 (one-tailed T-test). Bars: 100µm.

Adult HGF tg mice were analyzed by echocardiography and compared with controls. No difference was found between single transgenics (silent HGF and α-MHC-tTA) and wild-type mice (Table 1). At 100 days of age, HGF tg mice had a significantly higher left ventricle end systolic diameter (LVESD), in comparison with controls (Table 1 and Figure 4A) and the fractional shortening (FS) was significantly reduced (Table 1), suggesting that an extra-dose of HGF is unfavourable for cardiac contractility. To distinguish between prenatal and postnatal effects of HGF stimulation, DOX was not administered to pregnant mothers and suckling progeny and weaned pups received DOX starting from postnatal day 1 (prenatal HGF tg mice). At 113 days of age, also prenatal HGF tg mice showed increased LVESD and reduced FS (Table 1). This indicated that the extra-dose of HGF during prenatal life was critical to produce the systolic defect and that suppression of HGF expression after birth could not rescue contractile function.

**Table 1:** Echocardiography of HGF tg mice.

	wild-type	HGF tg	prenatal HGF tg	silent HGF	α-MHC-tTA
	(n = 11)	(n = 20)	(n = 6)	(n = 9)	(n = 6)
**FS**	0.49±0.08	0.34±0.09*	0.33±0.06*	0.46±0.07	0.46±0.13
**IVSTd (mm)**	1.133±0.181	1.172±0.281	1.259±0.176	1.128±0.116	1.200±0.291
**LVEDD (mm)**	3.772±0.402	4.052±0.484	3.970±0.473	3.861±0.371	3.732±1.140
**PWTd (mm)**	1.009±0.128	1.002±0.232	1.010±0.125	0.981±0.091	0.970±0.130
**IVSTs (mm)**	1.654±0.203	1.602±0.414	1.614±0.227	1.648±0.140	1.639±0.455
**LVESD (mm)**	1.948±0.435	2.663±0.504*	2.665±0.449*	2.113±0.456	2.149±0.915
**PWTs (mm)**	1.476±0.180	1.374±0.305	1.332±0.092	1.502±0.065	1.397±0.189
**h/r**	0.571±0.068	0.539±0.115	0.577±0.090	0.550±0.059	0.635±0.223
**HW (g)**	0.193±0.050	0.182±0.036	0.226±0.031	n.d.	n.d.
**BW (g)**	33.17±5.08	32.71±3.76	30.36±5.76	n.d.	n.d.
**HW/BW (g/g)**	0.006±0.001	0.006±0.001	0.007±0.001	n.d.	n.d.

Wild-type: littermate wild-type control; HGF tg: bitransgenic mice conceived in the absence of DOX; prenatal HGF tg: bitransgenic mice treated with DOX at birth and maintained in DOX thereafter; silent HGF and α-MHC-tTA: littermate single transgenics. FS, fractional shortening; IVSTd, interventricular septum thickness in end diastole; LVEDD, left ventricle end diastolic diameter; PWTd, posterior wall thickness in end diastole; IVSTs, interventricular septum thickness in end systole; LVESD, left ventricle end systolic diameter; PWTs, posterior wall thickness in end systole; h/r, heart rate; HW, heart weight; BW, body weight; HW/BW, heart weight/body weight ratio. n.d., not determined.

*p<0.005 versus wild-type (two-tailed T-test).

After echocardiographic analysis, bitransgenic animals and controls were sacrificed and heart/body weight ratios were measured (Table 1). No significant increase in heart weight and heart/body weight ratio was observed in any of the bitransgenic groups (HGF tg and prenatal HGF tg), relative to controls. Histology of adult bitransgenic hearts and trichrome staining did not reveal evidence of fibrosis or increased myocyte cross sectional area (CSA), compared with age-matched controls (Figure S2). Next, we analysed re-expression of β-MHC, a marker of cardiac dysfunction. Figure 4B shows higher levels of β-MHC in both HGF tg and prenatal HGF tg mice (p<0.05), while low protein level was found in age-matched controls. Furthermore, in the induced animals, in concomitance with re-expression of β-MHC, α-MHC appears to be decreased. In both HGF tg and prenatal HGF tg mice, Cx43 protein expression levels were significantly decreased also in the adult (Figure 4B, p<0.05). Cx43 is a component of gap junctions' channels, which contribute to communication between adjacent cells. To assess whether the reduced expression of Cx43 observed in HGF tg mice alters the permeability of gap junctions, we performed a functional test with Lucifer yellow. The fluorescent tracer showed significantly reduced propagation from wound to adjacent cells in HGF tg mice (Figure 4C, p<0.005).

### Induction of Constitutively Activated Met in Postnatal Cardiomyocytes Causes Reactivation of Fetal Gene Program and Cardiac Remodeling

We decided to extend observations to another gain-of-function model produced in our laboratory that, differently from the HGF tg mouse, allows activation of Met in the absence of the ligand and cannot be downregulated [22]. The Tpr-Met responder was crossed with the α-MHC-tTA transactivator to constitutively activate Met signalling in cardiomyocytes in a cell-autonomous manner. We found that expression of Tpr-Met in prenatal development was lethal shortly after birth in 100% of cases. Bitransgenic Tpr-Met mice were observed with an expected Mendelian ratio of 25% at E16 (n = 17), E18 (n = 20) and P1 (n = 21), while no viable bitransgenics were found alive at P4 (n = 90). Thus, constitutive activation of Met starting from prenatal cardiac development leads to death of pups after birth.

**Figure 5:**
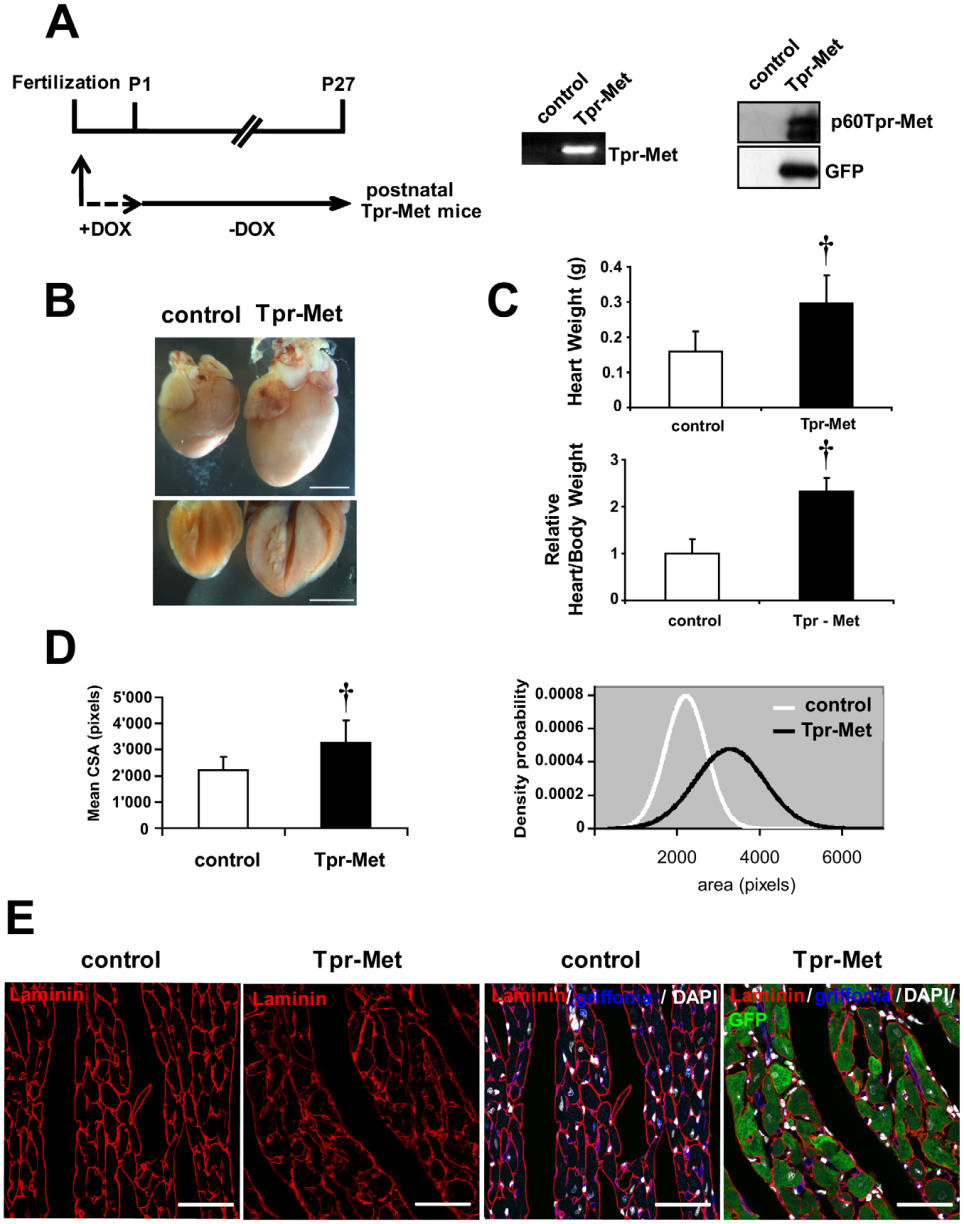
Induction of activated Met in postnatal cardiomyocytes leads to hypertrophy. (A) Experimental design (left), RT-PCR (middle) and immunoblot (right) of Tpr-Met expression in postnatal Tpr-Met mice (P27) with Doxycycline (DOX) suppression until birth, compared to controls. (B) Control and postnatal Tpr-Met hearts were analyzed under stereomicroscopy for comparison (upper panel). Four-chamber cut hearts are also showed (lower panel). Bars: 5 mm. (C) Significantly increased heart weight (upper graph) and heart/body weight ratio (lower graph) indicate cardiac hypertrophy in postnatal Tpr-Met mice (n = 6 animals per group). †p<0.005 vs control (two-tailed T-test). (D) Mean cross-sectional area (CSA) of ventricular cardiomyocytes is significantly higher in postnatal Tpr-Met compared to controls (left panel). n = 300 cells from 3 biological replicates per group. †p<0.005 vs control (two-tailed T-test). Distribution curves (right panel) of counted CSA show a shift to the right side in postnatal Tpr-Met mice as respect to controls. (E) Representative transversal sections of left ventricles show increased size in postnatal Tpr-Met cardiomyocytes. Left panels: laminin (red-surface). Right panels: quadruple overlay with laminin (red- surface), Griffonia (blue-endothelial), DAPI (white-nuclear) and GFP (green-intracellular). Bars: 35µm.

**Figure 6:**
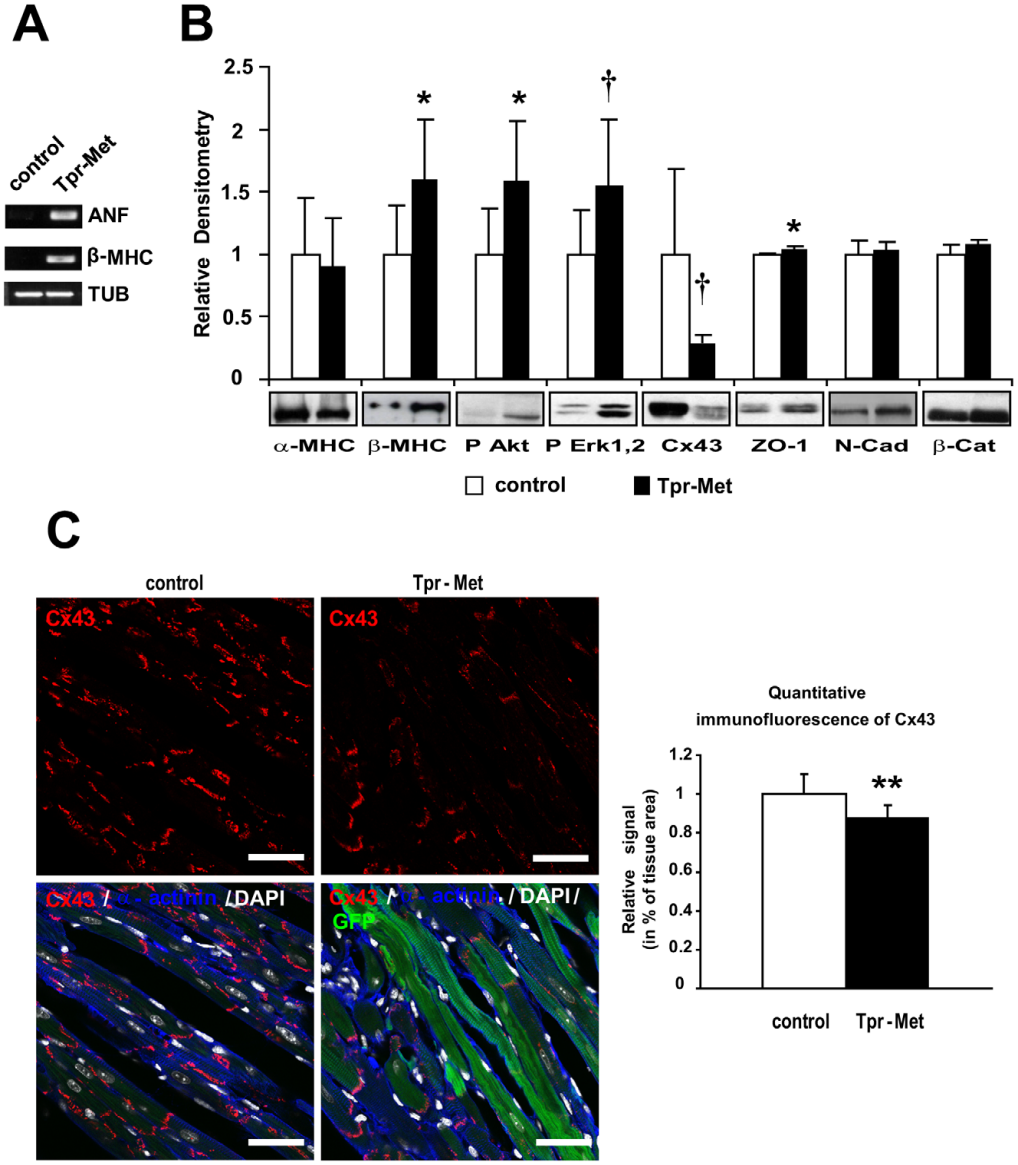
Tpr-Met expressing hearts show fetal gene re-expression and remodeling of Cx43. (A) Semi-quantitative RT-PCR analysis of controls and postnatal Tpr-Met mice (P27) showed re-expression of ANF and β-MHC mRNA. Tubulin is used as loading control. (B) α to β isoform switch of MHC, increased phosphorylation of downstream Akt and Erk1,2 and strongly decreased Cx43, mild increase of ZO-1 and normal N-Cadherin and β-Catenin levels in postnatal Tpr-Met vs control hearts, analyzed by Western blot. Densitometric quantification normalized on Akt loading control and representative blots below graphs are shown. n = 10 mice for each group. *p<0.05 and †p<0.005 vs control (two-tailed T-test) (C) Left panels: representative confocal immunofluorescence images of left ventricle sections from postnatal Tpr-Met mice showed decreased staining of Cx43 (red), compared to controls (upper panels). Bottom panels: quadruple overlay with Cx43: red; α-actinin: blue; DAPI: white-nuclear; GFP: green-intracellular; Bars: 35µm. Quantification of Cx43 staining was performed with ImageJ. n = 6 controls and n = 3 postnatal Tpr-Met mice.

To overcome the early lethality of Met hyperactivation and to evaluate effects of permanent Met activation in postnatal cardiomyocytes, Tpr-Met mice were conceived and delivered in the presence of DOX to suppress expression of Tpr-Met during *in utero* development. The day following birth, DOX was removed from drinking water to allow expression of the transgene (Figure 5A) in postnatal Tpr-Met mice. In the Tet-Off system, there is no expression at day 0 after the removal of DOX, minimal expression from days 3 to 7 and maximal expression after day 11 [23]. Transgene expression was verified at both mRNA and protein levels at P27 (Figure 5A). Postnatal Tpr-Met died at ∼4 weeks after birth with signs of congestive heart failure (n = 6), including lung oedema, alopecia, ascytes, dyspnea and lethargy (Figure S3). Animals were sacrificed at day 27 and the heart weight and the heart/body weight ratio were measured. At visual inspection under the stereomicroscope, Tpr-Met+ hearts showed impressively enlarged ventricles, with thick ventricular wall and interventricular septum (Figure 5B). Postnatal Tpr-Met mice had significantly increased heart mass (0.296 g±0.080 vs control 0.160 g±0.056, p<0.005) and heart/body weight ratio (2.3 fold increase, p<0.005), indicating a marked ventricular hypertrophy (Figure 5C). Consistent with weight measurements, histology of cardiac tissue and cross sectional area measurements from postnatal Tpr-Met mice demonstrated increased ventricular cardiomyocyte size, compared to controls (p<0.005; Figure 5D,E and Figure S4). Moreover, cardiomyocytes from postnatal Tpr-Met mice were not only characterized by an increased area, but also by a high variability in size compared to cardiomyocytes from control animals (Figure 5D,E and Figure S4), possibly due to variegated expression of Tpr-Met protein in cardiomyocytes. Altogether, these results suggest that the hypertrophic phenotype in Tpr-Met heart may not only involve an increase in cross sectional area but also myocyte disarray and heterogeneous volume of the cardiomyocyte population. Only modest signs of fibrosis were found (data not shown). Reactivation of fetal genes, frequently associated with hypertrophy and heart failure, was also observed, with increased ANF and β-MHC mRNA, as detected by semi-quantitative RT-PCR (Figure 6A). When analyzed in Western blot, the α- to β isoform switch of MHC, typical of heart failure, was detected in postnatal Tpr-Met hearts (Figure 6B). Strong activation of both phospho-Akt and phospho-Erk1,2 was observed (Figure 6B). Notably, a strong reduction of Cx43 levels was seen in postnatal Tpr-Met hearts by Western blot and immunofluorescence analysis (Figure 6B,C and Figure S5), indicating remodeling of gap junctions. In contrast, mild upregulation of ZO-1 and no difference in N-Cadherin and β-Catenin proteins were detected in Western blot (Figure 6B). The change of Cx43 pattern was detectable already at P15, preceding the onset of hypertrophy (data not shown).

## Discussion

In this article, first we demonstrate that during the early postnatal period of rapid growth, neonatal cardiomyocytes express the Met receptor *in vivo* and can respond to exogenous HGF by activating downstream signalling. These findings are corroborated *in vitro* in H9c2 cardiomyoblast cell line. Second, we show that an extra-dose of HGF expression (and consequent Met activation) acts on neonatal cardiomyocytes by influencing both proliferating and differentiating parameters. The finding that Erk1,2 phosphorylation is significantly enhanced in HGF-stimulated cardiomyoblasts *in vitro* and in neonatal cardiomyocytes of bitransgenic mice *in vivo* suggests that the mitogenic activity of HGF arises from MAPK activation, in concordance with a previous report [24]. Notably, transition from hyperplastic to predominant hypertrophic growth in the mouse has been estimated to happen during the first week after birth [25]. In our HGF gain-of-function model, at seven days post birth, we found increased proliferation and decreased sarcomeric protein levels, indicating that the transition between the plastic phase and the mature, post-plastic phase was temporally delayed.

It has been shown that cardiomyocyte cell proliferation is accompanied by a decrease of cell-cell communication [26], [27] and Cx43 has been proposed to contribute to contact inhibition of cell growth. Accordingly, together with increased proliferation during postnatal age, we observed markedly low levels of Cx43. This is in line with previous studies demonstrating that HGF inhibits intercellular communications via gap junctions in hepatocytes and keratinocytes [28], [29], where it acts as a powerful mitogen. While the HGF effect on the contractile machinery was limited in time, Cx43 protein levels were maintained low lifelong. Cx43 was found to be downregulated even in adult mice with HGF expression suppressed the day following birth. This suggests that an imperfect organization of cell-cell communication during development triggers a functional defect that cannot be subsequently reversed during adult life. Remodeling of gap junctions has been observed in a variety of cardiomyopathic conditions, including hibernating myocardium, infarction and dilated cardiomyopathy [30]. Observations in ischemic and hibernating heart disease have led to the thesis that gap junction remodeling may contribute not only to defects in electrical signal conduction, but also to impairment of contractile force [31]. To date, evidence linking gap junction remodeling with ventricular dysfunction has been correlative, with the exception of studies done on chimeric mice, composed of variable mixtures of Cx43-null and wild-type cells throughout all tissues of the body [32]. These mice showed no morphological abnormalities, myocardial fibrosis or hypertrophy, but developed significant systolic dysfunction. Mechanistically, in our gain-of-function model, regional dysregulation of Cx43 in cardiomyocytes, due to excessive activation of the HGF/Met system during development, could lead to a loss of coordinated contraction throughout the heart wall and, consequently, local increased systolic wall stress and ventricular dysfunction. We propose that alteration of growth specifically in the fetal/neonatal heart and/or of intercellular communication may prime it to develop an increased susceptibility to disease.

The inducible character of our HGF mouse model demonstrates that prenatal development is the specific stage influenced by activation of Met signalling. In fact, at 3 months of age our HGF tg mice developed a contractile defect, even when HGF expression was suppressed after birth. This result is consistent with the finding that the endogenous Met receptor is physiologically downregulated in terminally differentiated cardiomyocytes, making the system insensitive to further HGF stimulation. The high susceptibility of prenatal age to Met stimulation is further confirmed by the fact that expressing Tpr-Met instead of HGF starting from prenatal age was lethal soon after birth. The Tpr-Met fusion protein lacks the extracellular, transmembrane and juxtamembrane domains of Met receptor and has gained the Tpr dimerization motif, which allows constitutive and ligand-independent activation of the kinase. The loss of juxtamembrane sequences necessary for the negative regulation of kinase activity and receptor degradation prolongs duration of Met signalling [22]. For this reason, the Tpr-Met model represents a very strong gain-of-function model of Met activation and yields an exacerbated cardiac defect with respect to the HGF model. Hypoxia and growth restriction are well described causes of developmental origin of cardiovascular disease. Ligand-independent Met overexpression is induced by the hypoxia inducible factor-1 (HIF-1) [33] and by depletion of von Hippel-Lindau protein, which is responsible for suppressing HIF-1 levels during normoxia [34]. Hypoxia and Met itself are responsible for inducing an adaptive process known as invasive growth through which the organism attains homeostasis, in particular foraging for supplies for cell survival, such as oxygen and glucose. We propose that an alteration of Met signalling, such as HGF/Met overexpression, giving a message of nutrition or oxygen restriction, could mimic the molecular effect of these environmental cues. Such a trigger could induce an adaptive response pathway, which would be maintained over time through an epigenetic footprint. It will be interesting to investigate whether elevated Met receptor levels have a putative role in the etiology of hypoxia-initiated cardiac disease.

Sustained activation of Tpr-Met in postnatal cardiomyocytes (1 to 4 weeks) leads to increased cross sectional area of cardiomyocytes, reactivation of fetal gene program, increased cardiac mass and, ultimately, to lethal congestive heart failure at P28. Thus, the constitutive activation of Tpr-Met gives to the cell a signal of growth, which, in terminally differentiated cardiomyocytes, results in switching on a hypertrophic program. Both Ras/RAF/MEK/ERK and Akt pathways, which are downstream to Met and Tpr-Met, are known to be involved in the growth promotion and protection of cardiomyocytes from apoptosis. However, their contribution in defining “physiological” versus “pathological” growth is still controversial. In the HGF model, the low level of Met receptor, which cannot be superinduced by HGF stimulation in the terminally differentiated cardiomyocyte, cannot shift the equilibrium to hypertrophic growth. Interestingly, in the postnatal Tpr-Met model we found only mild signs of interstitial fibrosis, albeit pathological hypertrophic growth is usually associated with scar tissue formation. This finding confirms the antifibrotic action of HGF/Met activation, which has been demonstrated in a variety of tissues, including the heart [35].

Tpr-Met expression induced a dramatic decrease in Cx43 protein levels in postnatal cardiomyocytes. This reinforces the concept that Met receptor activation acts negatively on cell-cell communication, albeit the precise mechanism by which this suppression is mediated awaits elucidations. Evidence is in favour of the view that both formation and maintenance of gap junctions is critically dependent on the presence of correct mechanical stabilization [36]. The interdependence between mechanical and electrical junctions seems to be unilateral, since the absence of Cx43 does not change the structure of intercalated discs with respect to adherens junctions, as shown in an animal model with cardiac-specific conditional knockout of Cx43 [37]. This seems to be the case of our study, since both Tpr-Met and HGF-activated Met signalling in cardiomyocytes maintain connexins in a remodeled state, with Cx43 being downregulated at the protein level and removed from end-to-end intercalated discs. Meanwhile proteins constituting adherens junctions show no quantitative abnormalities, though a slight increase of cell adhesion proteins has been observed in heart of postnatal Tpr-Met mice. The changes in Cx43 pattern distribution were seen at a fairly early stage in the disease progress, suggesting that Cx43 may be an early indicator of cardiac stress.

In conclusion, our mouse models support the idea that HGF/Met stimulation promotes cardiomyocyte growth. Although other studies have suggested that HGF may have a beneficial function in pathological conditions, such as ischemic injury, there are no experimental evidences in the current study to demonstrate that enhancement of HGF/Met signalling is favourable in a physiological setting. On the other hand, excessive HGF/Met signalling in prenatal period may raise adverse effects and might be linked to the pathogenesis of progressive cardiac disease.

## Supporting Information

Figure S1 Neonatal HGF tg hearts show no morphological defects. Haematoxylin-eosin staining of four-chamber cut sections of P7 control (left) and HGF tg (right) hearts. Bars: 2mm.
(0.88 MB TIF)

Figure S2 No signs of fibrosis nor hypertrophy were found in adult HGF tg mice. (A) Trichrome staining does not show fibrosis in either control or littermate HGF tg mice at 4 months of age. (B) Cross-sectional area of myocytes was not different between control and HGF tg mice at 4 months of age (green-surface: laminin; green-intracellular: GFP; blue-nuclear: DAPI; red-endothelial: Griffonia). Bars: 50 µm (A); 75µm (B).
(3.14 MB TIF)

Figure S3 Postnatal Tpr-Met mice at P27 display signs of congestive heart failure. (A) Tpr-Met mice exhibit dyspnea and lethargy. Extensive oedema and haemorrhage of Tpr-Met lungs shown by haematoxylin and eosin staining of lung tissue (B), stereomicroscopy inspection (C) and lung weight measurement (D), compared to littermate controls. n = 4 animals per group. * p<0.01 vs control (two-tailed T-test). Bars: 20mm (A); 100mm (B); 5mm (C).
(1.23 MB TIF)

Figure S4 Single immunofluorescence stainings of quadruple overlay shown in Figure 5E: Laminin (red-surface), Griffonia (blue-endothelial), DAPI (white-nuclear), GFP (green-intracellular) and 4 colours merge. Bars: 35 µm.
(2.67 MB TIF)

Figure S5 Single immunofluorescence stainings of quadruple overlay shown in Figure 6E: Cx43 (red), α-actinin (blue), DAPI (white-nuclear), GFP (green-intracellular) and 4 colours merge. Bars: 35 µm.
(2.66 MB TIF)

Table S1 Primers used throughout the study.
(0.31 MB PDF)

Table S2 List of antibodies used in this study.
(0.59 MB PDF)
