# Supplementary figures and images for: Activated Met Signalling in the Developing Mouse Heart Leads to Cardiac Disease
Source: PLoS One. 2011 Feb 9;6(2):e14675. doi: 10.1371/journal.pone.0014675 (PMC3036588; doi:10.1371/journal.pone.0014675)

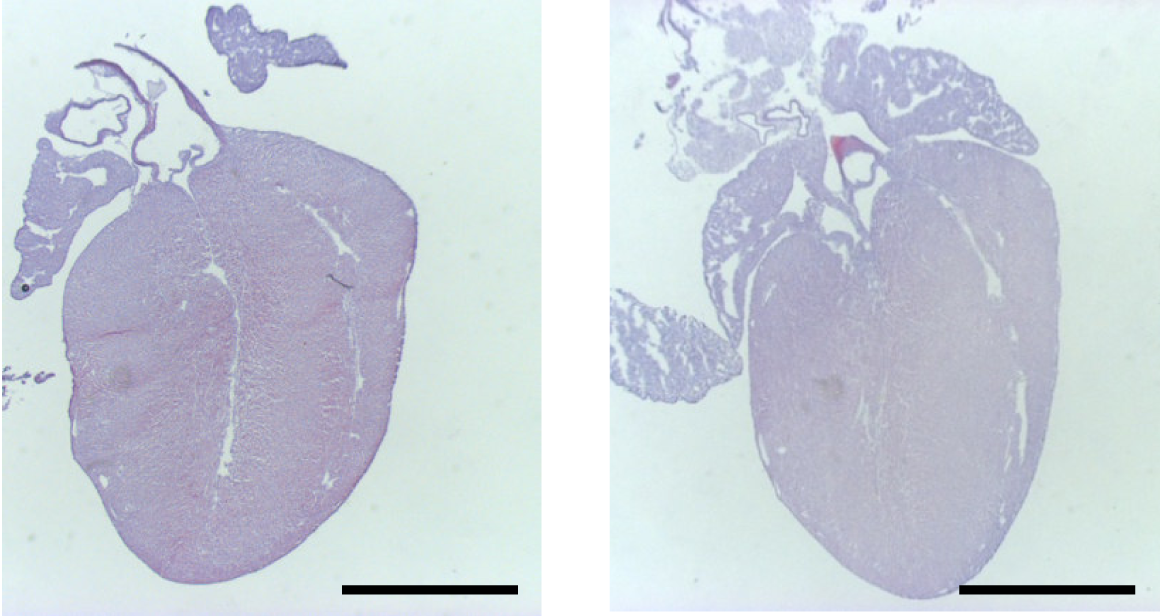

Supplement: Figure S1 — Neonatal HGF tg hearts show no morphological defects. Haematoxylin-eosin staining of four-chamber cut sections of P7 control (left) and HGF tg (right) hearts. Bars: 2mm. (0.88 MB TIF) [file pone.0014675.s001.tif]

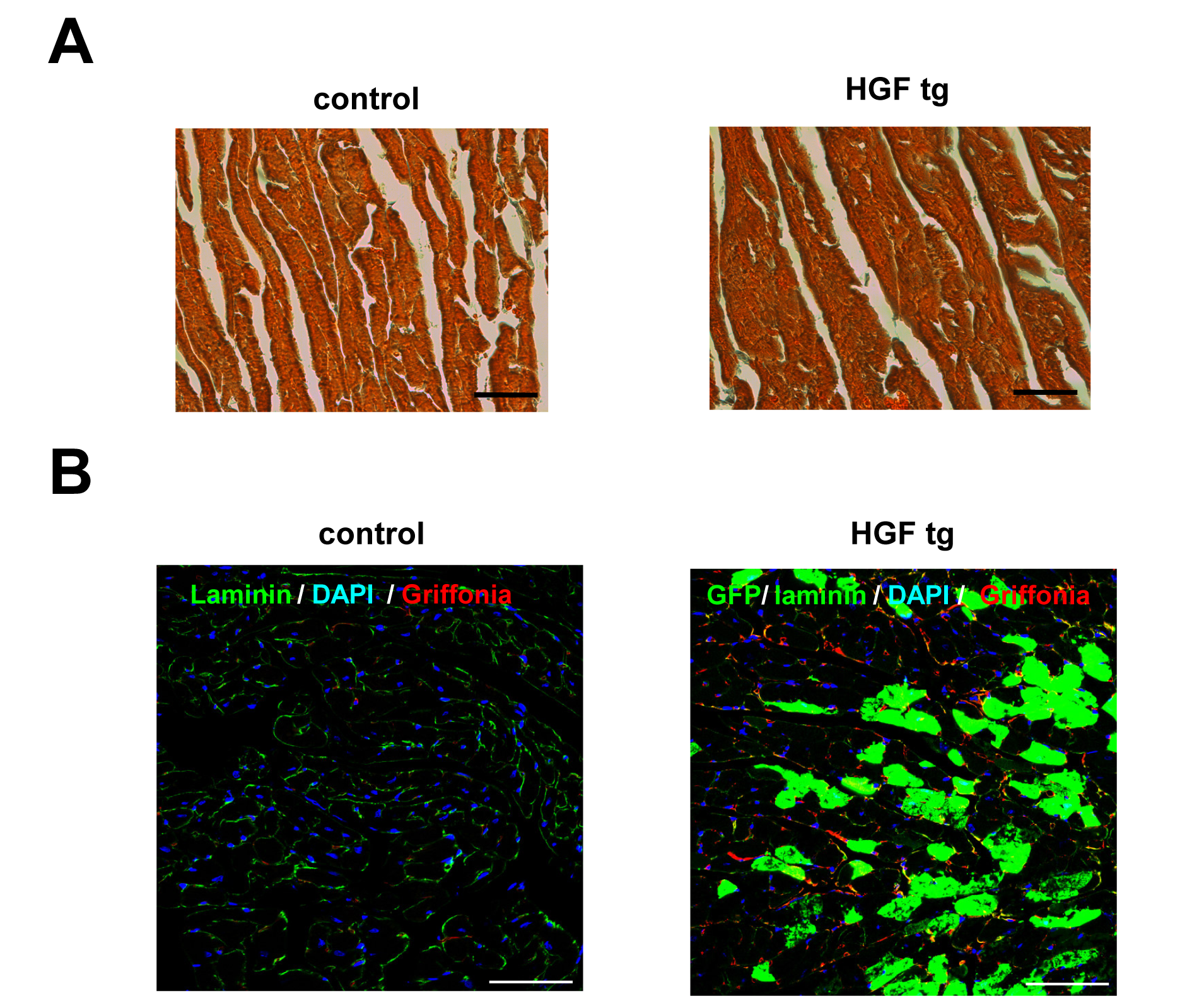

Supplement: Figure S2 — No signs of fibrosis nor hypertrophy were found in adult HGF tg mice. (A) Trichrome staining does not show fibrosis in either control or littermate HGF tg mice at 4 months of age. (B) Cross-sectional area of myocytes was not different between control and HGF tg mice at 4 months of age (green-surface: laminin; green-intracellular: GFP; blue-nuclear: DAPI; red-endothelial: Griffonia). Bars: 50 µm (A); 75µm (B). (3.14 MB TIF) [file pone.0014675.s002.tif]

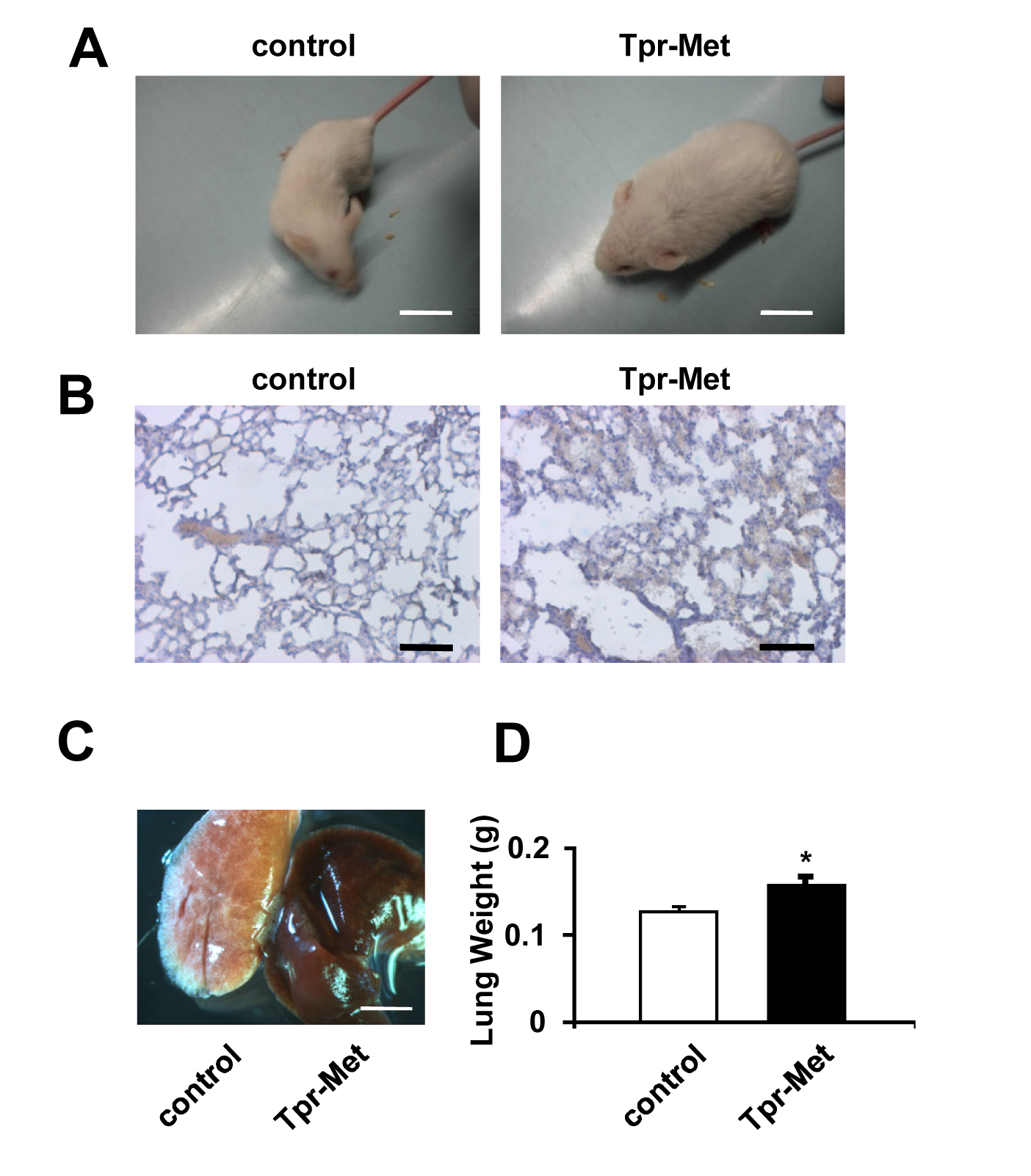

Supplement: Figure S3 — Postnatal Tpr-Met mice at P27 display signs of congestive heart failure. (A) Tpr-Met mice exhibit dyspnea and lethargy. Extensive oedema and haemorrhage of Tpr-Met lungs shown by haematoxylin and eosin staining of lung tissue (B), stereomicroscopy inspection (C) and lung weight measurement (D), compared to littermate controls. n = 4 animals per group. * p<0.01 vs control (two-tailed T-test). Bars: 20mm (A); 100mm (B); 5mm (C). (1.23 MB TIF) [file pone.0014675.s003.tif]

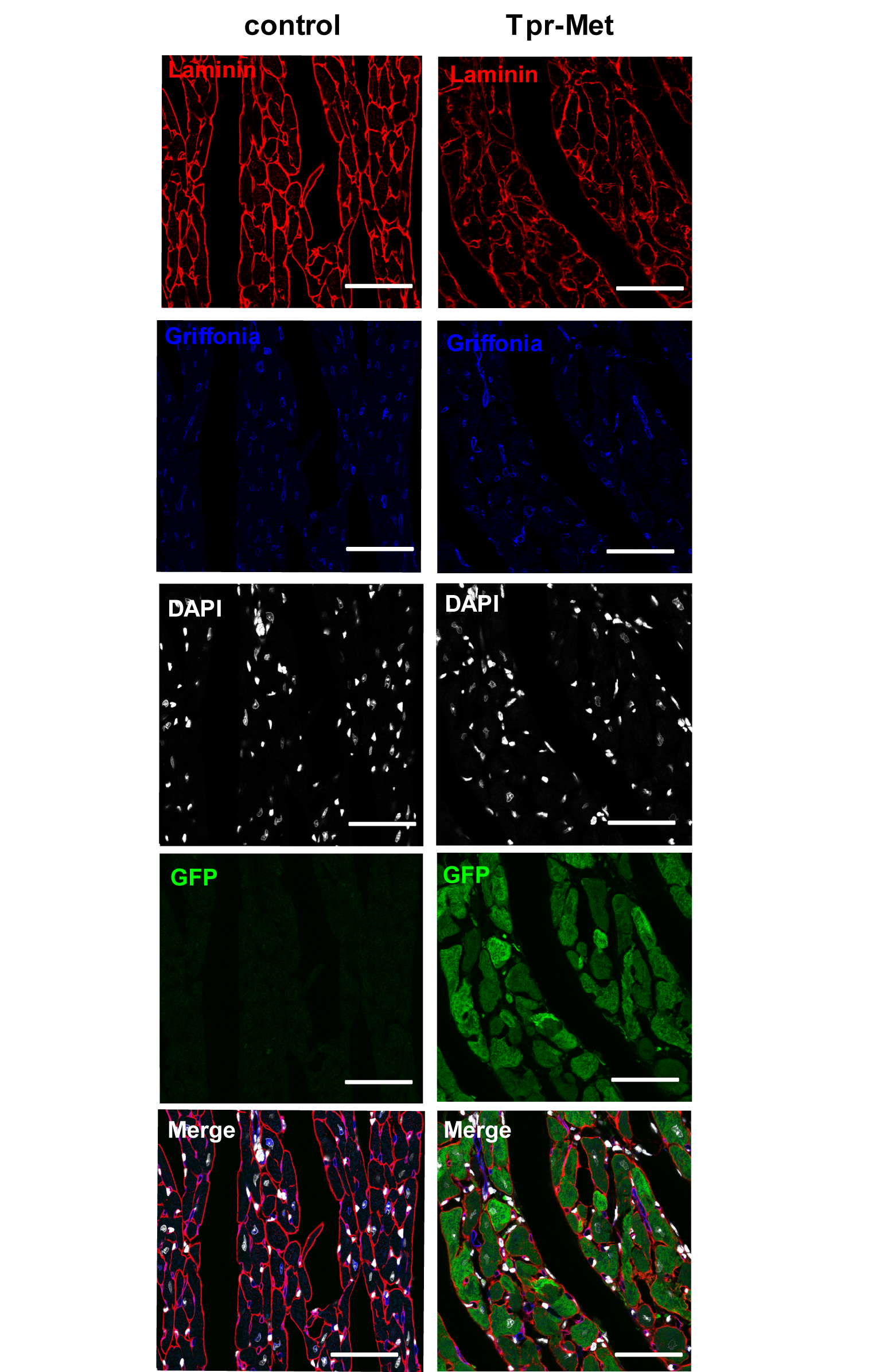

Supplement: Figure S4 — Single immunofluorescence stainings of quadruple overlay shown in Figure 5E: Laminin (red-surface), Griffonia (blue-endothelial), DAPI (white-nuclear), GFP (green-intracellular) and 4 colours merge. Bars: 35 µm. (2.67 MB TIF) [file pone.0014675.s004.tif]

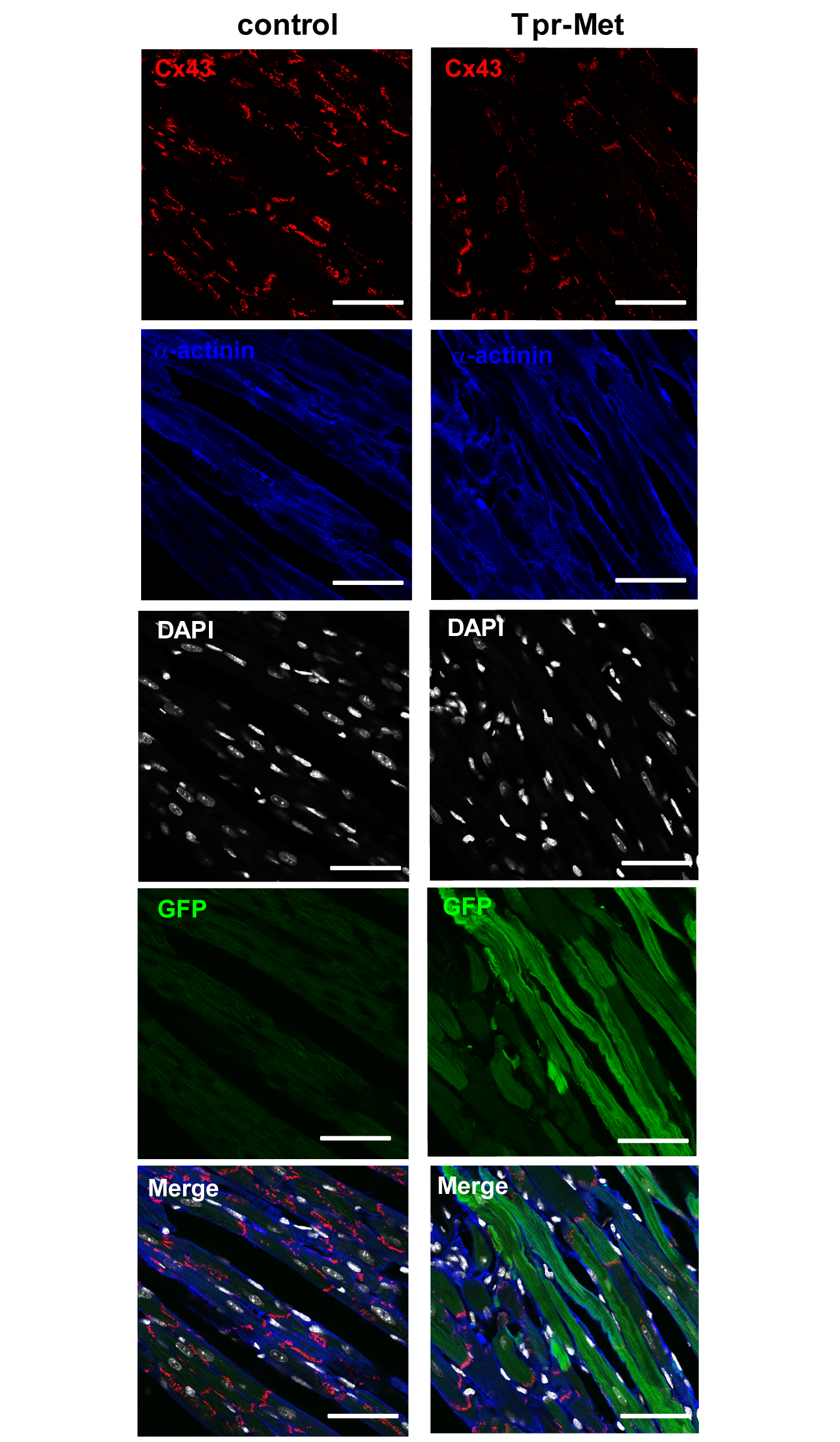

Supplement: Figure S5 — Single immunofluorescence stainings of quadruple overlay shown in Figure 6E: Cx43 (red), α-actinin (blue), DAPI (white-nuclear), GFP (green-intracellular) and 4 colours merge. Bars: 35 µm. (2.66 MB TIF) [file pone.0014675.s005.tif]
