# Supplementary material for: Activated Met Signalling in the Developing Mouse Heart Leads to Cardiac Disease
Source: PLoS One. 2011 Feb 9;6(2):e14675. doi: 10.1371/journal.pone.0014675 (PMC3036588; doi:10.1371/journal.pone.0014675)
Supplement: Table S1 — Primers used throughout the study. (0.31 MB PDF) [file pone.0014675.s006.pdf]

Table S1. Primers used throughout the study.

Assay	Forward	Reverse
HGF cDNA construct	5'TTGCACGCGTCCACCAT GATGTGGGGGACCAAAC	5'TTACACGCGTGTTAACTTACTTT CCAAGTCGGTTCATCTCTATGTCT GTATACAACTTGTATGTCAAAA
Genotyping α-MHC tTA	5'AAGTGATTAACAGCGCA TTAGAGC	5'TTCAAGGCCGAATAAGAAGGCT GG
Genotyping HGF responder	5'ACCTGCAATCCTGGATA ACTTTGT	5'GGTCCCCAAACTCACCCTGAAG TTCTC
Genotyping Tpr-Met responder	5'AGA GGA GCC CCTCCT TAT CC	5'GGT CCC CAA ACT CAC CCT GAA GTT CTC
Semi quantitative PCR HGF	5'ACCTGCAATCCTGGATA ACTTTGT	5'GGTCCCCAAACTCACCCTGAAG TTCTC
Semi quantitative PCR Tubulin	5'GCTGTGATTGCCTGCAA GGC	5'TCGAGACGTGGGAATGGCA
Semi quantitative PCR Tpr-Met	5'AGAGGAGCCCCTCCTTA TCC	5'GGTCCCCAAACTCACCCTGAAG TTCTC
Semi quantitative PCR ANF	5'CAAACATCAGATCGTGC CCCGA	5'TTTGCTTTTCAAGAGGGCAGAT
Semi quantitative PCRα-MHC	5'GCCAACACCAACCTGTC CAA	5'TGCAAAGGCTCCAGGTCTGAGG GC
Real-time PCR exogenous HGF	5'GACATAGAGATGAACCG ACTTGGAA	5'CACCCTGAAGTTCTCAGTCTAG AGA
Real-time PCR FAM exogenous HGF Taqman probe	5'ACGCGTGTTAACTTAC	
Real-time PCR total HGF	5' CACCACTTGGGAGTATT GTGC	5'GGGACATCAGTCTCATTCACAG
Real-time PCR FAM total HGF Taqman probe	5'GCTCACAG	
