# Supplementary material for: Activated Met Signalling in the Developing Mouse Heart Leads to Cardiac Disease
Source: PLoS One. 2011 Feb 9;6(2):e14675. doi: 10.1371/journal.pone.0014675 (PMC3036588; doi:10.1371/journal.pone.0014675)
Supplement: Table S2 — List of antibodies used in this study. (0.59 MB PDF) [file pone.0014675.s007.pdf]

Table S2. List of antibodies used in this study.

**Primary Antibodies for WB & IF:**			
Kindly given by S. S.	α-MHC	1:1000	clone BAG5
	Troponin I	1:1000	clone Ti4
	Troponin T	1:1000	clone RVC2
Sigma	β-MHC	1:1000	M8421
	Cx43	1:5000; 1:300 for IF	C6219
	α-tubulin	1:5000	T5168
	Laminin	1:1000 for IF	L8271
	BrdU	1:100	BU33
	α-actinin	1:1000 for IF	A7811
Santa Cruz Biotechnology	Erk2	1:1000	C14
	p140Met	1:1000; 1:300 for IF	SP260
	p60Tpr-Met	1:1000	C-12
Cell Signaling	P p38	1:1000	4631
	P Erk 1,2	1:1000	20G11
	P Akt	1:1000	9271s
	Akt	1:1000	9272
Zymed	N-Cadherin	1:1000	clone 3B9
	ZO-1	1:1000	61-7300
Molecular Probes	GFP	1:2000	A11122
Vector Laboratories	Rhodamine Griffonia	1:300 for IF	RL-1102
BDTransduction Laboratories	β-Catenin	1:1000	610153
R&D	HGF	1:500	AF294-NA
Open Biosystem	GAPDH	1:4000	TAB1001
Novocastra	Ki67	1:300 for IF	NCL-Ki67p
**Horseradish Peroxidase-Conjugated Secondary Antibodies for WB:**			
Amersham	Goat anti-mouse IG		31430
	Goat anti-rabbit IG		31460
**Secondary Antibodies for IF:**			
Molecular Probes	Alexa Fluor 488	1:500	A11034
	Alexa Fluor 488	1:500	A11029
	Alexa Fluor 546	1:500	A11030

	Alexa Fluor 546	1:500	A11035
	Alexa Fluor 647	1:500	A21244
Sigma	Mouse Cy3	1:500	C 2306
